# RhoA and Rac1 as Mechanotransduction Mediators in Colorectal Cancer

**DOI:** 10.1002/adbi.202400626

**Published:** 2025-01-30

**Authors:** Sharda Yadav, Sanjaya KC, Mark A. T. Blaskovich, Cu‐tai Lu, Alfred K Lam, Nam‐Trung Nguyen

**Affiliations:** ^1^ Queensland Micro‐ and Nanotechnology Centre Nathan Campus Griffith University Brisbane QLD 4111 Australia; ^2^ Institute of Molecular Bioscience The University of Queensland St Lucia QLD 4067 Australia; ^3^ School of Medicine and Dentistry Griffith University Southport QLD 4222 Australia

**Keywords:** colorectal cancer (CRC), mechanical strain, mechanotransduction, RhoA and Rac1, tumor microenvironment

## Abstract

Colorectal cancer (CRC) remains a leading cause of cancer‐related deaths, creating an urgent need for innovative diagnostic solutions. Mechanobiology, a cutting‐edge field that investigates how physical forces influence cell behavior, is now revealing new insights into cancer progression. This research focuses on two crucial players: RhoA and Rac1, small yet powerful proteins that regulate the structure and movement of cancer cells. RhoA controls cell adhesion and migration, while Rac1 drives cell movement and invasion. As CRC tumors grow and reshape the colon's mechanical environment, these pathways become disrupted, accelerating cancer progression. Examining the level of RhoA and Rac1 in CRC clinical samples under mechanical strain reveals their potential as diagnostic markers. Tracking the activity of these proteins can unlock valuable insights into cancer cell dissemination, offering new avenues for understanding and diagnosing CRC. This approach holds promise for earlier detection and better outcomes by offering key insights for more effective diagnostic strategies.

## Graphical abstract

Analysing RhoA and Rac1 protein levels in Colorectal cancer (CRC) samples under mechanical strain highlights their potential as diagnostic markers. Monitoring their activity could offer valuable insights into how cancer spreads, paving the way for new approaches to better understand and diagnose colorectal cancer.

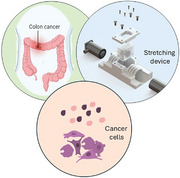

## 1 Introduction

Colorectal cancer (CRC) is one of the most prevalent and lethal malignancies worldwide, ranking as the third most common cancer and the second leading cause of cancer‐related deaths.^[^ ^1^ ^]^ Despite advancements in screening and treatment, the prognosis for advanced stages of CRC remains poor, demanding a deeper understanding of its underlying mechanisms to improve therapeutic strategies.^[^ ^2^ ^]^ Understanding cancer cells interaction within themselves and the tumor microenvironment has a role in elucidating some of these mechanisms. The tumor microenvironment is a complex and dynamic entity, composed of cancer cells, stromal cells, immune cells, and an extracellular matrix (ECM).^[^ ^3^ ^]^ Among the various factors influencing cancer progression, mechanobiology has emerged as a pivotal research area.^[^ ^4^ ^]^

Mechanobiology focuses on understanding how mechanical parameters and forces within the tumor microenvironment affect the behavior and progression of cancer cells. Mechanobiology explores how cells sense, generate, and respond to mechanical stimuli, encompassing a wide range of processes including cell adhesion, migration, proliferation, and differentiation.^[^ ^5^ ^]^ These processes are critical in the context of cancer, as they contribute to tumor growth, invasion, and metastasis.^[^ ^3^, ^6^ ^]^ In the context of CRC, the mechanical environment within the colon and rectum tissues undergoes significant alterations due to tumor growth and the remodeling of surrounding ECM. These mechanical changes can influence cancer cell signaling pathways, contributing to tumor progression and metastasis.^[^ ^7^ ^]^ The gastrointestinal tract is naturally exposed to various mechanical forces such as peristalsis, which can affect tissue homeostasis and cellular behavior.

One of the fundamental elements of mechanotransduction is a family of small nucleotide guanosine triphosphates (GTPases), including RhoA and Rac1.^[^ ^8^ ^]^ These molecular switches are pivotal in regulating the cytoskeleton, a dynamic network of fibers that provides structural support to cells and is crucial for various cellular processes.^[^ ^9^ ^]^ The cytoskeleton, composed of actin filaments, microtubules, and intermediate filaments, maintains cell shape, facilitates intracellular transport, and enables cellular movement. RhoA and Rac1 specifically play central roles in controlling cell shape, motility, and interactions with the ECM.^[^ ^10^ ^]^ RhoA is particularly known for its role in the formation of stress fibers and focal adhesions, which are essential for cell adhesion and migration. Stress fibers are actin filament bundles that generate contractile force, allowing cells to exert mechanical tension in their environment.^[^ ^11^ ^]^ Focal adhesions are complex structures where the actin cytoskeleton is anchored to the ECM, providing the necessary links for cells to adhere to their substrate and sense mechanical cues.^[^ ^12^ ^]^

Rac1, in contrast, is involved in the formation of lamellipodia and membrane ruffles, which are critical for cell movement. Lamellipodia are sheet‐like protrusions that extend from the leading edge of migrating cells, driven by the polymerization of actin filaments pushing the plasma membrane forward.^[^ ^13^ ^]^ Membrane ruffles are dynamic, wave‐like extensions of the plasma membrane that contribute to cell motility and the ability of cells to navigate through their environment.^[^ ^14^ ^]^ Through these mechanisms, Rac1 facilitates cell migration and invasion, which are crucial for various physiological processes as well as pathological conditions such as cancer.^[^ ^12^ ^]^

In the context of cancer, RhoA and Rac1 are often implicated in the regulation of key processes such as epithelial‐to‐mesenchymal transition (EMT), invasion, and metastasis.^[^ ^15^ ^]^ EMT allows epithelial cells to lose their cell adhesion properties and acquire migratory and invasive characteristics, enabling them to disseminate from the primary tumor and establish secondary tumors in distant organs.^[^ ^16^ ^]^ RhoA's regulation of stress fibers and focal adhesions contributes significantly to the reorganization of the cytoskeleton during EMT, facilitating changes in cell shape and motility that are critical for cancer progression.^[^ ^17^ ^]^ The involvement of Rac1 in the formation of lamellipodia and membrane ruffles further aids in the invasive behavior of cancer cells, allowing them to traverse the extracellular matrix and invade surrounding tissues.

In CRC, understanding how RhoA and Rac1 respond to mechanical stress is essential for deciphering their mechanobiological roles. Mechanical stress in tumors arises from factors such as tumor expansion, ECM alteration, and therapeutic interventions. These stresses could activate signaling pathways altering the gene expression, protein function, and cellular behavior.^[^ ^18^ ^]^ Investigating RhoA and Rac1 expression and activity under mechanical strain in CRC tissue samples offers valuable insights into cancer progression.^[^ ^19^ ^]^ This research could identify novel biomarkers for disease progression and therapeutic targets, with potential strategies focusing on modulating RhoA and Rac1 activity to disrupt cancer cell dissemination and improve patient outcomes.

This study investigates the expression patterns of RhoA and Rac1 under mechanical strain in post‐surgical CRC clinical samples to elucidate their roles in cancer progression and identify therapeutic targets. Clinical samples reflect tumor complexity, including genetic mutations, cell diversity, and the native microenvironment, offering more translatable insights than cell lines. Examining mechanobiology in vivo captures the heterogeneity and mechanical context of CRC, aiding the identification of biomarkers and predictive tools for treatment response. By validating findings in clinical samples, this research bridges laboratory studies and clinical applications, paving the way for improved therapeutic strategies and patient outcomes

## 2 Results

**Figure 1:**
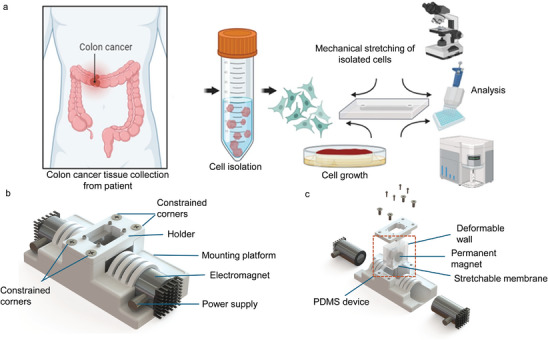
a) Schematic illustration of the assay for quantifying protein biomarkers in colorectal cancer cells. The cells were isolated from colon cancer tissue obtained post‐surgery from a patient. The cells were seeded on PDMS and subjected to mechanical stretching. After stretching, the cells were chemically lysed, and the supernatant was collected to quantify the released protein markers. Along with the morphological analysis of the stretched cells, the quantification of protein markers was performed using ELISA and flow cytometry. b) Experimental setup of the cell stretching platform c) Exploded view of the cell stretching platform and the polydimethylsiloxane (PDMS) device.

Evaluating the expression of protein markers, particularly Rho GTPases linked to carcinogenesis and various cancers, we have demonstrated that overexpression of RhoA and Rac1, members of the Rho GTPase family, is associated with cancer progression. In previous studies, we have demonstrated the overexpression of these markers in cell lines of breast cancer^[^ ^20^ ^]^ and liver cancer.^[^ ^21^ ^]^ In this study, we are expanding the application of these markers to colon cancer. We studied the overexpression of the biomarkers and their variations using tumor tissue from patients with colorectal cancer obtained post‐surgery. The results will confirm that mechanical stimulation not only induces overexpression of these markers in cancer cells but can provide enough information to differentiate between the clinical properties of colon cancer. Cancer cells were isolated from colon cancer to investigate this mechanism. The clinical cancer cells and control cells from cell lines were subjected to mechanical stretching, Figure 1a.

By applying mechanical stimulation to cancer cells from tissues, we aimed to mimic the dynamic mechanical environment within the human body and to observe its effect on the expression levels of protein biomarkers. This approach allows for validating our findings in a clinical context, demonstrating that mechanical stress affects the expression of carcinogenesis‐related protein markers in human cancer tissues.

In summary, both cancer tissue‐derived cells and control cancer cells were cultured until they reached confluence. Subsequently, the cells were transferred onto a polydimethylsiloxane (PDMS) device. Once the cells had adhered to the deformable PDMS membranes, mechanical stress was applied through cyclic stretching (Figure 1b). This process involved applying an input voltage of 27 V to both actuators, producing an average homogeneous cyclic strain of 1.38 ± 0.021% over the central region of the membrane. Force optimization was performed, ranging from 0.5 to 1 N with increments of 0.5 mN.^[^ ^22^ ^]^

### 2.1 Hematoxylin and Eosin (H&E) Stain on Cancer Cells

**Figure 2:**
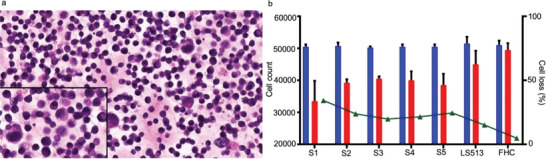
a) Photograph of H & E stain of cancer cells isolated from the clinical tumor tissue collected from colorectal cancer patients obtained post‐surgery. The Hematoxylin stain highlights the cell nuclei in a deep purple color, enabling clear visualization of the nuclear morphology. The Eosin stain shows a pink coloration to the cytoplasm. b) Bar graph shows total cell counts before (blue) and after (red) stretching, *n* = 3. The percentage of cell loss is determined by dividing the number of stretched cells by the total number of unstretched cells and multiplying the ratio by 100.

The staining method was employed to confirm that the cells extracted from the tumor tissue of patients with colon cancer were indeed cancerous and not normal cells. Following isolation from the patient tissue, cells were cultured and subsequently validated using H&E staining. As illustrated in Figure 2a, H&E staining showed a significant presence of cancer cells within the isolated cell population. The Hematoxylin stain, which specifically binds to nucleic acids, highlighted the cell nuclei in a deep purple color, enabling clear visualization of the nuclear morphology. In contrast, the Eosin stain, which binds to cytoplasmic components, imparted a pink coloration to the cytoplasm.^[^ ^23^ ^]^ This contrast allowed us to distinguish between the cellular components effectively, confirming the cancerous nature of the extracted cells.

H&E staining provided a detailed view of the cellular structure, revealing characteristic features of cancer cells such as increased nuclear‐to‐cytoplasmic ratio, prominent nucleoli, and irregular nuclear borders^[^ ^24^ ^]^ (Figure 2a). The zoomed sections of the figures provide a clearer view of the prominent nuclei, which appear in a deep purple color, along with an increased nuclear‐to‐cytoplasmic ratio. These observations were critical in validating the cells derived from the colorectal cancer tissue were malignant, ensuring the accuracy of our subsequent analyses and experiments.

### 2.2 Qualitative Analysis by General Microscopy and Morphology

We have previously demonstrated, cellular rearrangements as one of the effects after stretching breast and liver cancer cells. Furthermore, these cells showed noticeable morphological differences and created distinct cell‐cell connection structure.^[^ ^25^ ^]^ The purpose of this study was to evaluate whether mechanical strain had a direct effect on primary cells taken from colon cancer patients. The same mechanical stretching platform was applied for strain the cells recovered from colorectal cancer tissue. After stretching, we performed a microscopic analysis of the cells, allowing for the observation of their responses to external stress and the reorganization of their cytoskeleton. Additionally, we noted cell loss following the stretching process (Figure 2b). In our previous studies with breast and liver cancer cell lines, we noted the reconstruction and reorganization of actin stress fibers. In the present study, we compared clinical samples (labeled S1, S2, S3, S4, and S5) with colon cancer cell line LS513 and non‐cancer colon cell line, FHC. Primary cells directly derived from living tissues provide a more accurate in vivo model, preserving the characteristics of the original tissue. Primary cells maintain natural heterogeneity with a lower risk of genetic mutations when compared to the immortalized cell lines. Furthermore, these cells maintain more accurate physiological regulation. Many cellular pathways, such as cell signaling and differentiation, are regulated in a manner more like that in the original tissue.

**Figure 3:**
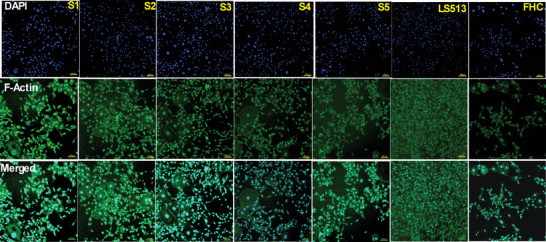
The representative images (x 10) of fluorescence staining of the five clinical cancer cells (S1, S2, S3, S4, and S5), cancer cells (LS513), and non‐cancer cells (FHC) after stretching. Cell nuclei are labeled with DAPI (blue), and actin is labeled with ActinGreen (green). Scale bar corresponds to 100 µm.

Figure 3 shows that sample S1 exhibited clustering, while S2 and S3 demonstrated strong cell connections and the formation of cell clusters. This indicates how individual cancer cells sense and transmit physical forces to and from neighboring cells. Also, S2 and S3 cells induced the rearrangement of actin microfilaments, suggesting a high level of intercellular communication and mechanical signaling within the population of cancer cells. Samples S4 and S5 also showed significant cluster formation. However, after stretching, cells in S1 and S5 began detaching more quickly than in other samples. The pathological stage of cancer plays a significant role in how cancer cells interact with shear forces and adhere to blood vessel walls. The behavior of the cytoskeleton and the cell's ability to metastasize is closely linked to the stage of tumor progression. S1, S4, and S5 are stage II cancers, while S2 and S3 are advanced‐stage tumors. In early‐stage tumor cells, the cytoskeleton is relatively stable and might not exhibit significant re‐organization in response to shear forces. In contrast, advanced‐stage cancer cells show considerable cytoskeletal re‐organization, making them more motile and better able to adapt to shear forces.^[^ ^26^ ^]^ This re‐organization helps advanced‐stage cancer cells become more invasive and enhances their ability to withstand mechanical stress, promoting metastasis.

We calculated the percentage of cell loss after stretching, Figure 2b. The sample S1 exhibits a significantly higher amount of cell loss at 34% compared to the other samples. Specifically, S2 shows 23%, S3 shows 19%, S4 shows 21%, and S5 shows 24% cell loss. In contrast, the cell line LS513 demonstrates only 15% cell loss. This difference in cell death can be the inherent sensitivity of primary cells and intact stress response pathways, including apoptosis (programmed cell death). These pathways can be more easily triggered in primary cells under stress, leading to increased cell death. On the other hand, cell lines often have mutations or alterations in these pathways, making them less responsive to stress. These genetic changes can disrupt normal apoptotic processes, making the cell lines less responsive to stress. As a result, cell lines are generally more resilient under the same conditions that would induce significant cell death in primary cells.

Furthermore, the non‐neoplastic colon epithelial cells (FHC cell line) show only 4% cell loss. This stark difference between cancerous and non‐cancerous cells can be attributed to the rigidity of the cell membrane. Cancer cells tend to have more rigid cell membranes, resulting in less tolerance to mechanical stretching. In contrast, non‐cancerous cells have more elastic cell membranes, providing them with a higher tolerance to mechanical stretching.^[^ ^26^, ^27^ ^]^ Additionally, the biochemical signaling pathways in non‐cancerous cells are intact, allowing them to react to external stress via an autocrine feedback mechanism. This feedback mechanism is often disrupted in cancer cells, leading to higher susceptibility to cell death under stress.^[^ ^28^ ^]^

### 2.3 Biomarkers Recovery from the Cells

All viable cells from the experiment were harvested for the quantification of RhoA and Rac1 markers. These protein markers are predominantly located in several key areas within the cell. Both are often associated with the inner surface of the plasma membrane upon activation by guanosine triphosphate (GTP) binding, where they interact with various downstream effectors to reorganize the actin cytoskeleton, essential for cell shape maintenance, motility, and adhesion. In their inactive guanosine diphosphate (GDP)‐bound states, RhoA and Rac1 reside in the cytoplasm, bound to guanine nucleotide dissociation inhibitors (GDIs), preventing their activation and interaction with downstream effectors.^[^ ^29^ ^]^ This cytoplasmic localization allows for rapid activation and translocation to the membrane in response to extracellular signals. Additionally, both RhoA and Rac1 have been found in the nucleus, where they influence gene expression and other nuclear functions by interacting with nuclear proteins and regulatory elements. As intracellular kinases, RhoA and Rac1 require cell lysis, a process that breaks down the cell membrane, to release them into the sample for measurement.

For cell lysis, we used NP‐40, a mild surfactant effective for studying cytoplasmic and membrane‐bound proteins. As described in the “Materials and Methods” section, the supernatant from lysed cells was collected and analyzed using ELISA to quantify RhoA and Rac1 levels and flow cytometry to assess their expression at the single‐cell level.

### 2.4 Overexpression of Protein Biomarkers After Mechanical Strain

Previous studies have demonstrated that protein biomarkers are elevated in breast and liver cancer cells in response to mechanical stretching. However, these results were primarily based on the cells obtained from the American Type Culture Collection (ATCC).^[^ ^20^, ^21^ ^]^ In this study, we sought to validate these findings using clinical samples obtained from patients who had undergone surgery. Specifically, we measured the relative expression levels of protein biomarkers RhoA and Rac1 before and after applying mechanical strain to the five clinical samples. We compared these samples with colon carcinoma LS513 cells and non‐cancer colon epithelial cells (FHC) as controls. RhoA has been implicated in multiple cancers including breast carcinoma, liver carcinoma, ovarian carcinoma, and gastric carcinoma. Our investigation focused on the expression of RhoA in clinical cells collected from human colorectal carcinoma specimens and the cells purchased from ATCC. We confirm that RhoA is overexpressed in the clinical cancer samples compared to non‐cancer control samples. Additionally, we correlated the RhoA expression with several factors including age, gender, stage, metastasis, and invasion. Our findings revealed that RhoA expression is significantly associated with the clinical stage of colorectal cancer. This suggests that higher levels of RhoA expression may be indicative of more advanced stages of colorectal cancer. However, we did not find a significant correlation between RhoA expression and the age of the patients.

**Figure 4:**
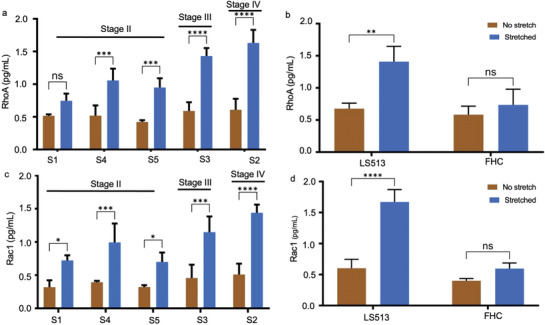
Clinical evaluation of the protein biomarkers before and after applying the mechanical strain. a) Quantitative measurement of RhoA activity in clinical cancer samples (S1, S4, S5, S3, and S2) pre and post‐stretching. b) Quantitative measurement of RhoA activity in the cancer cell (LS513) and non‐cancer cell (FHC) pre‐ and post‐stretching. c) Quantitative measurement of Rac1 activity in clinical cancer sample (S1, S4, S5, S3, and S2) pre and post stretching. d) Quantitative measurement of Rac1 activity in the cancer cell (LS513) and non‐cancer cell (FHC) pre‐ and post‐stretching.  The error bar represents the standard deviation of the experiments (*n* = 3). Statistical significance was determined by pairwise comparisons between two conditions (cancer stages) using a two‐way ANOVA test with Tukey's multiple comparisons test.

In this experiment, cells were subjected to mechanical stretching for 2 h. Following this mechanical intervention, the cells underwent chemical lysis to release their intracellular contents. The levels of the protein biomarkers RhoA and Rac1 were then quantified using Enzyme‐Linked Immunosorbent Assay (ELISA). Our results confirmed that mechanical strain leads to a significant increase in the expression of these biomarkers in clinical samples, consistent with the observations made in ATCC‐derived cell lines. As shown in Figure 4a, there was a significant increase of RhoA markers in the patient sample when compared to the control non‐neoplastic cell line, FHC (Figure 4b, Table 1). Histopathological analysis revealed that three samples were of stage II, one stage III, and one stage IV (Table 2). As expected, the relative expression of RhoA was much higher in stage IV cancer samples, followed by stage III, and II respectively. The incidence of RhoA increased with the advancing tumor stage. Specifically, we observed the following changes in RhoA expression of clinical samples: The expression observed in Stage IV, (Sample S2) increased from 0.5 pg mL^−1^ before stretching to almost 1.55 pg mL^−1^ after stretching; Stage III (Sample S3) increased from 0.5 to 1.4 pg mL^−1^; similar phenomena regarding the cancer stage dependency of the RhoA expression was observed in stage II (Sample S1) increased from 0.5 to 0.7 pg mL^−1^; (Sample S4) increased from 0.5 to 1.0 pg mL^−1^; and (Sample S5) increased from 0.3 to 0.9 pg mL^−1^. In the cancer cell line LS513, RhoA levels increased from 0.6 pg mL^−1^ before stretching to 1.4 pg mL^−1^ after stretching. In contrast, the control non‐cancer cell FHC showed a modest increase from 0.5 to 0.6 pg mL^−1^, which was not statistically significant. These results indicate a pronounced and consistent upregulation of RhoA in response to mechanical stretching in both clinical cancer samples and cancer cells; with significantly higher increases observed in cancerous cells when compared to non‐cancer cells. It is easy to discriminate the RhoA expression between the non‐cancer control and stage II cancer samples. These data suggest that our assay is capable of quantifying differential expression patterns of RhoA in different stages of colon cancer. Statistical significance was determined by pairwise comparisons between two conditions (cancer stages) using a two‐way ANOVA test.

**Table 1:** *p‐values* obtained using a two‐way ANOVA test comparing the control and different stages of colon cancer after stretching.

Comparison	Rho A	Rac 1
Non‐cancer vs Stage II	0.99	0.81
Non‐cancer vs Stage III	<0.005	<0.05
Non‐cancer vs Stage IV	<0.0005	<0.0005
Stage II vs Stage III	<0.005	0.051
Stage II vs Stage IV	<0.005	<0.005
Stage III vs Stage IV	0.51	0.23

**Table 2:** Clinical information on colon cancer patients.

Patient Number	Age^a)^	Gender^b)^	Site^c)^	Size^d)^	Grade^e)^	Stage^f)^
P1	61	M	Sigmoid colon, Rectum	55	2	II
P2	70	F	Transverse colon, Ascending colon	20	2	IV
P3	76	M	Descending colon	37	2	III
P4	80	M	Sigmoid colon, Ascending colon	37	2	II
P5	59	M	Rectum	95	2	II

^a)^ In years.

^b)^ M, male; F, female.

^c)^ Site of tumor.

^d)^ Tumor size (in mm) after histological analysis.

^e)^ Grade of the cancer.

^f)^ Stage if the cancer (stage I, stage II, stage III, stage IV).

In alignment with previous findings, our study also detected significant levels of Rac1 in clinical cancer samples and cancer cells compared to non‐cancer cells (Figure 4c,d). Notably, Rac1 expression in clinical cancer samples exhibited a marked increase following mechanical stretching. Specifically, stage IV (Sample S2) increased from 1.4 pg mL^−1^, stage III (Sample S3) increased from 0.4 to 1.2 pg mL^−1^, stage II (Sample S1 and S4) increased from 0.3 to 0.7 pg mL^−1^, and from 0.4 to 1.0 pg mL^−1^ respectively. Similarly, in the cancer cell line LS513, Rac1 levels surged from 0.5 before stretching to 1.5 pg mL^−1^ after stretching. In contrast, the non‐cancer cell line showed no significant difference, with only a modest increase from 0.4 to 0.6 pg mL^−1^ following mechanical stretching.

Our study demonstrates that mechanical stretching significantly upregulates the expression of the protein biomarkers RhoA and Rac1 in clinical cancer samples and cancer cells, with a clear correlation between increased biomarker levels and advanced cancer stages. This upregulation was most pronounced in Stage IV samples and least in Stage II samples, with similar trends observed in the cancer cell line LS513. In contrast, non‐cancer cells showed only modest, non‐significant changes in biomarker levels. These findings suggest that mechanical stress plays a crucial role in enhancing the expression of RhoA and Rac1 in cancer cells, highlighting the potential of these biomarkers in assessing cancer progression and the mechanical stress response.

### 2.5 Scanning Electron Microscopy (SEM) Analysis of Cancer Cells

We conducted SEM analysis on cancer cells isolated from the patient's post‐surgical tissue samples. The cells were cultured as previously described and then subjected to mechanical strain to investigate the effects of stretching on their structure. SEM was employed to observe and analyze the morphological changes induced by mechanical stretching. As part of the SEM preparation, the cells were fixed, dehydrated, and coated with a conductive material, following standard protocols to preserve and visualize the structural alterations caused by the applied mechanical strain. The cells were analyzed both before and after the application of mechanical strain. This is to provide a comparative understanding of how stretching affects cancer cell structure.

**Figure 5:**
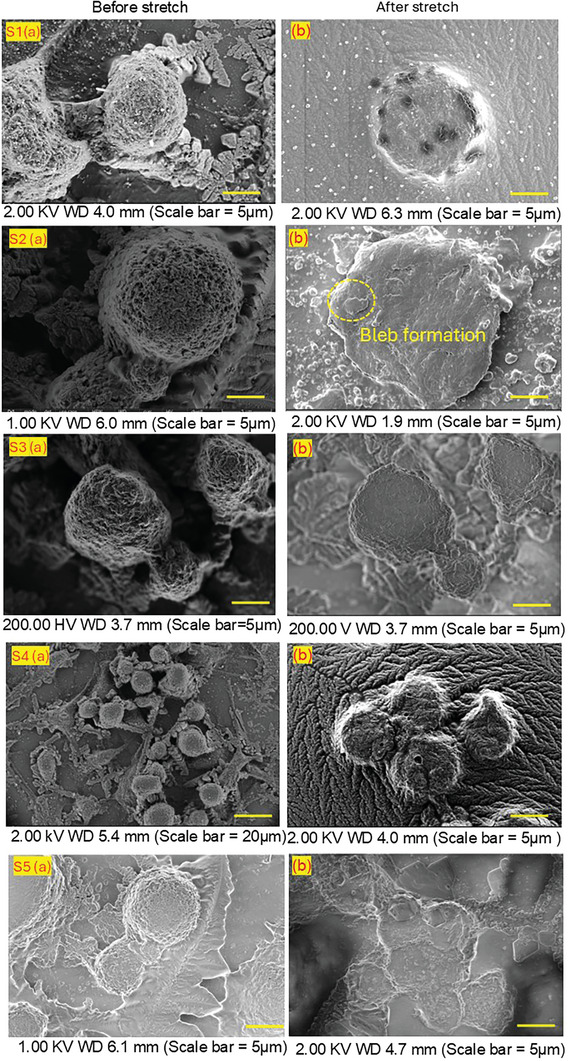
SEM images of cancer cells isolated from the clinical tumor tissue collected from colorectal cancer patients obtained post‐surgery. The images show cancer cells from five different clinical samples (S1, S2, S3, S4, and S5) both before a) and after application b) of mechanical strain. After stretching, several changes were observed; the cells appeared slightly flattened, with an increase in surface roughness, suggesting possible cytoskeletal reorganization. Additionally, the samples showed the formation of blebs on the cell surface.

As depicted in Figure 5, significant changes were observed across the different samples. In sample S1 (stage II), we noted distinct alterations in the surface structures of the cells. The cells appeared slightly flattened, with an increase in surface roughness, indicating potential reorganization of the cytoskeleton. Additionally, there was a notable increase in the presence of stress fibers, which are key indicators of the cell's response to mechanical forces. This suggests that mechanotransduction pathways were activated, leading to the reorientation of the cytoskeleton in response to the strain. In contrast, samples S2 (stage IV) and S3 (stage III) exhibited more pronounced flattening and irregular cell shapes. The irregularity in shape and increased flattening are likely direct consequences of enhanced mechanotransductive signalling, which triggers cytoskeletal remodelling. These samples also revealed the formation of blebs on the cell surface. Bleb formation is a typical response to changes in membrane tension and cytoskeletal dynamics, driven by mechanotransduction. Such features are often associated with the early stages of apoptosis, which can be induced by mechanical strain in cancer cells.^[^ ^4^ ^]^ SEM imaging effectively captured these characteristic blebs, providing insight into the cells' stress responses. Similarly, samples S4 and S5 (stage II) displayed marked changes, including the stress fibers and a pronounced reorientation of the cells, coupled with a more flattened structure.

When cancer cells are subjected to mechanical strain, their response in terms of bleb formation, surface flattening, and irregularity can vary depending on the stage of cancer.^[^ ^30^ ^]^ Mechanical strain can amplify the mechanotransductive signals that lead to distinct morphological changes in cells, which evolve as the cancer progresses.^[^ ^31^ ^]^ These changes are highly dynamic and evolve with the advancing stages of cancer, reflecting the increasing complexity of tumor biology. For example, in early‐stage cancer, cells show moderate surface flattening with minor shape irregularities, with minimal and localized bleb formation, indicating initial cytoskeletal disruptions. Research on human renal carcinoma cells demonstrated that shock wave‐induced cytoskeletal deformation can trigger bleb formation.^[^ ^32^ ^]^ Furthermore, blebbing has been closely linked to critical cancer processes, such as cell locomotion and tumor metastasis, underscoring its role in disease progression.^[^ ^30^ ^]^ As cancer progresses to the intermediate stage as in S1, S4, and S5, these cells undergo more pronounced flattening and significant irregularity in shape, with minimal and localized bleb formation suggesting increased cytoskeletal stress and enhanced cell motility. In advanced‐stage cancer such as S2 and S3, cells display extensive surface flattening, severe irregularity, and numerous blebs. These reflect profound cytoskeletal disruption not only supports invasive behavior but may also indicate apoptosis due to excessive strain. For instance, these morphological changes, driven by mechanotransduction pathways, are crucial indicators of tumor aggressiveness, offering valuable insights for diagnosis and treatment strategies.

These findings highlight the intricate relationship between mechanical forces, cytoskeletal integrity, and cancer cell morphology. The interplay between cytoskeletal disruptions and bleb formation serves not only as a marker of tumor stage but also as a potential mechanism driving metastasis and invasive behavior in cancer cells.

### 2.6 Overexpression of Biomarkers at the Cellular Level

We performed flow cytometry analysis on the cells isolated from cancer tissue samples. Cells were prepared as per procedures followed for cell isolation, culture, and stretching in previous sections.

**Figure 6:**
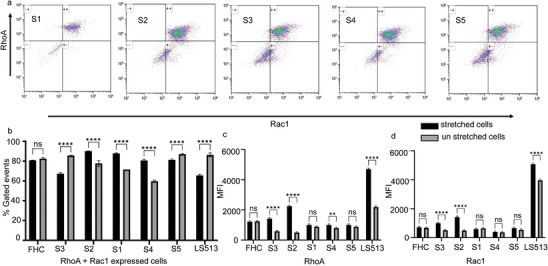
a) Density plot of flow cytometry data for Rac1 versus RhoA positive cells in clinical samples showing most of the cells express either RhoA or/and Rac1 post stretching. There is no correlation between the fraction of cells expressing these biomarkers and the disease stage or mechanical stress b). However, the levels of these biomarkers RhoA c) and Rac1 d), differs in different cancer stages and do respond to mechanical stretching for overproduction of these markers.

In the flow cytometry analysis, a detailed gating strategy was implemented to accurately isolate singlet cancer cells. This ensures that only individual cells were analyzed without the interference of doublets or cell clumps. After successfully gating for singlets, the expression of two critical biomarkers, RhoA and Rac1, was assessed (Figure 6a). The analysis revealed that a significant proportion of the cells expressed either RhoA, Rac1, or both, indicating the presence of these proteins across most of the cancer cell population (Figure 6b). However, we examined the Mean Fluorescence Intensity (MFI) to gain deeper insights into the relative expression levels. This quantitative analysis revealed a distinct difference in the expression levels of RhoA (Figure 6c) and Rac1 (Figure 6d) between stretched and unstretched cells, suggesting that the physical state of the cells may influence the expression of these key biomarkers.

As depicted in Figure 6c, the relative expression level of RhoA is significantly elevated in the LS513 cancer cell line following mechanical strain, in contrast to the FHC non‐cancer cell line, which does not exhibit any notable increase after stretching. This differential response highlights the unique mechanotransduction pathways activated in cancer cells, suggesting that RhoA plays a critical role in how cancer cells adapt to mechanical stress. When examining clinical samples, stage III and IV tumor samples (S3 and S2) show a marked increase in RhoA levels post‐stretching, compared to stage II samples (S1, S4, and S5). This pattern suggests that RhoA expression not only correlates with tumor progression but also intensifies as the cancer advances, reflecting the increasingly aggressive and resilient nature of higher‐stage tumors.

Similarly, as shown in Figure 6d, the relative expression of Rac1 is also elevated in advanced‐stage cancers compared to intermediate‐stage cancers. This consistent upregulation of both RhoA and Rac1 in more advanced tumors points to their potential as key biomarkers for cancer progression.

Flow cytometry analysis enabled the precise quantification of RhoA and Rac1 at the single‐cell level, offering a detailed, high‐resolution view of the cellular heterogeneity within tumor populations. The technique allows for the identification of subpopulations of cancer cells that may exhibit higher levels of these markers, providing insight into how different cells within the same tumor might contribute to its overall progression and resistance to treatment. By revealing these cellular‐level differences, flow cytometry helps to map the landscape of cancer progression more accurately. It shows how specific cell populations evolve as the disease advances, and how their expression profiles change in response to mechanical strain. This detailed understanding at the cellular level is critical for developing more effective stage‐specific treatments. In addition, it is useful for identifying new therapeutic targets that could potentially inhibit the cancer progression at its earliest stages.

## 3 Discussion

An incresaing number of studies have gradually highlighted the pivotal role of RhoA and Rac1 in cancer development and progression. These small GTPases, key regulators of cytoskeletal dynamics, cell motility, and proliferation have been recognized for decades as crucial players in the initial stage of malignancy and metastatic potential of various cancers. Although the role of RhoA and Rac1 in cancer progression is well‐established, it is interesting to further investigate how mechanical strain influences their expression and activity in CRC. Investigating these markers in CRC clinical samples subjected to mechanical stress provides deeper insights into the mechanobiological mechanisms that drive cancer progression. Such studies could reveal how mechanical forces within the tumor microenvironment influence the behavior of cancer cells, potentially leading to new therapeutic strategies targeting these mechanotransduction pathways in CRC.

Our data presents a detailed study of the role of mechanical stimulation on the expression of Rho GTPases, specifically RhoA and Rac1, in CRC. The overexpression of these protein markers, previously established in ATCC‐derived cell lines of breast and liver cancers, is confirmed in colon cancer cells of clinical samples. It has been achieved through various experimental approaches, including mechanical stretching of both clinical tumor samples and established cell lines. The findings highlight the significant impact of mechanical stress on cancer progression and the potential of Rho GTPases as biomarkers for assessing tumor stage and aggressiveness. The increase in RhoA and Rac1 levels observed after mechanical stretching in clinical samples supports the hypothesis that these markers are closely associated with the mechanical environment of the tumor. The correlation between biomarker expression and cancer stage, particularly the higher expression levels in advanced‐stage tumors, suggests that RhoA and Rac1 could serve as reliable indicators of tumor progression. The SEM analysis provided further insights into the cellular responses to mechanical stress. The morphological changes observed, such as cell flattening, increased surface roughness, and bleb formation, are indicative of cytoskeletal reorganization driven by mechanotransductive signalling. These changes were more pronounced in advanced‐stage cancer samples, reflecting the increased mechanosensitivity of these cells. The presence of stress fibers and the formation of blebs in response to mechanical strain suggest that the cytoskeleton plays a crucial role in mediating the cellular response to mechanical forces, which may contribute to the invasive potential of cancer cells.

Our flow cytometry analysis data provided crucial insights into the cellular mechanisms underlying cancer progression, particularly in response to mechanical strain. By implementing a rigorous gating strategy to isolate singlet cancer cells, we ensured the accuracy of our data, eliminating the potential interference from doublets or cell clumps. The subsequent assessment of RhoA and Rac1 expression across the cancer cell population revealed that these biomarkers are widely present, with a significant portion of cells expressing one or both proteins. More importantly, the examination of Mean Fluorescence Intensity (MFI) highlighted distinct differences in RhoA and Rac1 levels between stretched and unstretched cells, emphasizing the role of mechanical stress in modulating these key biomarkers.

The upregulation of RhoA and Rac1 in response to mechanical stress highlights the critical role of the tumor microenvironment in cancer progression. These biomarkers could serve as diagnostic and prognostic tools, potentially guiding the development of targeted therapies. SEM analysis further supports the impact of mechanical forces on cancer cell structure, suggesting that targeting mechanotransduction pathways could inhibit cancer cell motility and invasiveness. This emphasizes the need to consider mechanical factors in therapeutic strategies. While our study offers important insights into the role of mechanical stimulation in cancer progression, it's important to acknowledge certain limitations. The mechanical stretching model used may not fully replicate the complexity of the dynamic mechanical environment within the human body. Additionally, the sample size of clinical tumor tissues was limited, which may affect the generalization of our findings. Further studies with larger cohorts are needed to validate these results and explore the potential of RhoA and Rac1 as clinical biomarkers.

In conclusion, our study demonstrates that mechanical stimulation significantly enhances the expression of RhoA and Rac1 in colorectal cancer cells, with a clear correlation between biomarker levels and cancer stage. The morphological changes observed in response to mechanical stretching suggest that mechanotransduction plays a key role in cancer cell behavior, particularly in terms of cytoskeletal reorganization. These findings highlight the potential of RhoA and Rac1 as biomarkers for assessing tumor progression and the importance of considering mechanical factors in cancer treatment strategies.

## 4 Experimental Section

### Electromagnetic Actuated Device for Cell Stretching

The electromagnetic‐ actuated device for cell stretching, detailed in the previous publications, includes a mounting stage with electromagnets for cyclic strain, a holding clip, and a polydimethylsiloxane (PDMS) membrane with embedded NdFeB permanent magnets.^[^ ^22^, ^25^ ^]^ This device, designed for mechanobiology research, allowed cells to experience both static and cyclic stretching conditions. The PDMS membrane, with embedded magnets positioned 8 mm apart, deforms under magnetic forces to induce strain. The device is mounted on an electromagnetic stage using a custom clip that secures its orientation. The electromagnet, controlled by a programmable DC power supply, acts as the system's actuator. Initial investigations by Kamble et al. optimized parameters such as force, strain rate, and stretching conditions to simulate natural cellular environments, finding that an input voltage of 27 Volts for both actuators produced a strain of 1.38 ± 0.021%.^[^ ^22^ ^]^ These optimized parameters were adapted for the current experiment.

### Tumor Tissue Collection and Processing

Tissue samples from five patients diagnosed with colorectal carcinomas were obtained from the Gold Coast University Hospital with ethics approved by the Human Ethics Committee of Griffith University (GU Ref No: MSC/17/10/HREC). Before enrolling in the trial, all patients provided written informed consent. All colon cancer patients selected in this study were between the clinical stages II to IV. The age group of the patients range from 55 to 80 years (average age 70 years). The tumor tissue sample was collected in a sterile container with RNAlater (to prevent degradation of RNA) right after the surgery and transported on ice to maintain its viability.

### Isolation of Primary Cells from Tumor Tissue

**Figure 7:**
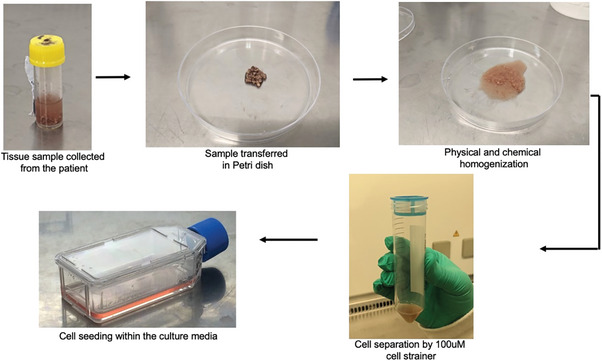
The procedure and process of cell extraction from fresh tumor tissue samples took 2.5–3 h. The tissues were first minced into small pieces. They were then transferred for enzymatic digestion at 37 °C with shaking. Any non‐digested tissue pieces were separated using a 100‐µm cell strainer. The extracted cells were immersed in cell culture media and immediately seeded in a culture flask.

Primary cells were isolated from tumor tissue following accepted guidelines (Figure 7). In a sterile environment, the tissue was rinsed with phosphate buffer saline (PBS) to remove blood or debris and cut into small pieces (1–2 mm) using a sterile surgical scalpel blade. The tissue pieces were transferred into a digestion solution containing enzymes (1 mg mL^−1^ Collagenase). The tissue was incubated in a shaking water bath at 37 °C for ≈2 h, with gentle agitation to help the enzymes digest the extracellular matrix and release single cells. After digestion, the cell suspension was filtered through a cell strainer (100 µm) to remove undigested tissue fragments. The filtered suspension was centrifuged at 300–400 *g* for 5 min to pellet the cells. The supernatant was discarded, and the cell pellet was resuspended in a culture medium. The centrifugation step was repeated to wash the cells, discarding the supernatant each time. The final cell pellet was resuspended in the culture medium and was transferred to a culture flask; incubated at 37 °C in a humidified incubator with 5% CO_2_. The culture medium was changed the next day to remove non‐adherent cells and debris. All the primary cells were used within five passages (P5) for the experiments.

### Validation of Cancer Cells by Cell Block Histology

The isolated cells were validated by cell blocking; Cells were grown in the flask and concentrated to a higher cell count. The cells were resuspended in PBS and centrifuged at 1000 × *g* for 5 min. The PBS was removed, and a few drops of human plasma and equal drops of thrombin were added to initiate the clot formation. The sample was mixed by tapping gently and allowed to clot. The clot entraps all the cells together. The samples were fixed overnight with formalin. The fixed clot was transferred in a lens paper and put inside the histology cassette and sent to the pathology laboratory for processing.

### Culture of Primary Clinical Cells

Five clinical sample cells extracted from the tumor tissue were cultured and maintained in DMEM/F12 medium supplemented with 10% foetal bovine serum (FBS) and 1% penicillin/streptomycin. The cells were grown in T75 flasks at 37 °C in a humidified environment with 5% CO₂ to allow the cells to adhere and start to proliferate. The culture medium was changed after 24 h to remove non‐adherent cells and debris and then every 2–3 days as needed. The cells were regularly monitored under a microscope to check for contamination and assess cell morphology and confluence. When the cells reached 70–80% confluence, they were sub‐cultured by trypsinising and reseeding at a lower density. During subculturing, the old culture medium was aspirated, and the cells were washed with PBS to remove residual serum. Trypsin‐EDTA solution was added to detach the cells, which were then neutralized with a culture medium containing FBS. The cell suspension was centrifuged at 300 × *g* for 5 min, and the pellet was resuspended in a fresh culture medium. The cells were reseeded into new flasks and placed back in the incubator.

### Culture of Immortal Control Cells

Human colorectal carcinoma LS513 and normal colon FHC cells were purchased from the American Type Culture Collection (ATCC). They were cultured and maintained in DMEM/F12 medium supplemented with 10% foetal bovine serum (FBS) and 1% penicillin/streptomycin. The cells were grown in T75 flasks at 37 °C in a humidified environment with 5% CO_2_.

### Seeding and Culturing Cells on the Deformable Membrane of the Electromagnetic Stretching Device

The electromagnetic device was sterilized using 80% ethanol and then rinsed three times with sterile 1× Hank's Balanced Salt Solution (HBSS), followed by 30 min of UV irradiation. Before seeding, the PDMS membrane was treated with 400 µL of DMEM‐F12 medium and incubated for 1 h to enhance biocompatibility. When the cancer cells in the T75 flask reached 80% confluence, they were harvested and counted using a hemocytometer. A total of 50,000 cells were seeded onto the PDMS membrane and incubated for 24 h to promote adhesion and growth. Afterward, the cultured cells were washed three times with HBSS and replenished with 400 µL of the medium. Mechanical strain was then applied as described in the subsequent section.

### Cyclic Stretching on the Cells

The PDMS membrane was secured onto the electromagnetic platform and placed in a 5% CO_2_ environment at 37 °C to maintain physiological conditions. Based on optimization works from the previous studies, the cells were exposed to a 1.4% strain at 0.01 Hz with a 50% duty cycle.^[^ ^22^, ^25^ ^]^ The strain applied to the membrane was characterized both experimentally and numerically. A voltage range of 1–30 Volts was applied to the actuator, and the deformation was captured using a digital camera (EO Edmund). The specified strain was administered for 2 h. After this, the cells were lysed to measure the levels of released biomarkers. The detailed cell lysis procedure is described in the following section.

### Cellular Fractionation

After the stretching cycles were completed, the cells were harvested by washing them three times and then treating them with trypsin to detach them from the membrane. The cells were then centrifuged, pelleted, and counted before undergoing chemical lysis. For lysis, a buffer was prepared with 1% Nonidet‐40 (NP‐40), 50 mm Tris‐HCl, and 20 mm ethylenediaminetetraacetic acid (EDTA) at pH 7.5. The cells were first washed with ice‐cold PBS before lysis following cell lysis buffer and incubated on ice for 30 min. Subsequently, the lysate was centrifuged at 5000 rotations per minute (RPM) for 5 min to separate the cell debris. The isolated pellet was rinsed with 1 mL of ice‐cold PBS. The supernatant, which contained the protein markers, was collected for further quantification by ELISA and stored at −20 °C.

### Quantification of RhoA and Rac1 Biomarkers

The levels of RhoA and Rac1 were measured using the RHOA ELISA Kit and RAC1 ELISA Kit from My BioSource, Inc., San Diego, CA, USA, respectively, in accordance with normal protocol. In brief, 100 µL of the lysed cell solution was added to each well of pre‐coated 96‐well plates. The plates were incubated for 1 h. Then, 100 µL of detection reagent A was added to each well, and the plates were incubated for another hour. After washing three times, 100 µL of detection reagent B was added, and the plates were incubated at 37 °C for 30 min. Following another three washes, 90 µL of horseradish peroxidase (HRP) substrate solution was added, and the plates were incubated for 20 min. The reaction was stopped by adding 50 µL of stop solution. A microplate reader (SpectraMax) at 450 nm was used to measure the color changes. The optical density (OD) readings were compared to a standard curve to quantify the concentrations of RhoA and Rac1 in the samples.

### Immunofluorescence

Standard immunofluorescence staining was employed to visualize the actin filaments and nuclei of cells seeded on the PDMS membrane. The process started with fixing the cells, both before and after the application of mechanical stretching, using 4% paraformaldehyde (PFA) for 30 min to preserve cellular structures. Following fixation, the cells were washed three times with HBSS to remove excess PFA and prepare the cells for staining. The cells were then stained using ActinGreenTM 488 and NucBlueTM ReadyProbeTM reagents from Thermo Fisher Scientific (Waltham, MA, USA), followed by a 30 min incubation at room temperature. Lastly, the stained cells were washed three times with HBSS to eliminate any unbound dye, ensuring clear and specific visualization of the actin filaments and nuclei under a fluorescence microscope.

### Image Analysis

The PDMS membrane containing the fixed and stained cells was carefully cut and placed directly onto a microscope slide. This preparation was essential for ensuring that the cells remained intact and properly oriented for imaging. High‐resolution images of the actin fibers and nuclei were captured using a fluorescent microscope (Nikon Eclipse Ti2), highlighting the cellular architecture and any changes resulting from the experimental conditions. ImageJ 1.47v was employed to analyze these images. The image processing software was developed by the National Institutes of Health (Bethesda, MD, USA).

### SEM Imaging

The cells were grown in a culture medium until they reached the desired confluency. Once they had reached confluency, the cells were stretched as required. The samples were then rinsed with PBS to remove residual media or debris. The samples were fixed by immersing them in a 4% PFA solution for 2 h at 4 °C. Following fixation, the samples were washed three times with Hanks’ balanced salt solution (HBSS) for 10 min each to remove any excess fixative. The samples were dehydrated through a graded ethanol series, sequentially immersing them in 30%, 50%, 70%, 90%, and 100% ethanol for 10–15 min each. The final dehydration step was performed twice in 100% ethanol to ensure complete dehydration. The dried samples were then mounted onto SEM stubs using carbon tape, ensuring that they were securely attached and correctly oriented for SEM imaging. Finally, the samples were placed into the SEM (Apreo 2S ThermoFisher) chamber. Images were acquired at various magnifications to capture detailed surface morphology and ultrastructure.

### Flow Cytometry Analysis

The cells were harvested and washed twice with 1×PBS by centrifugation at 500 × *g* for 5 min each time, discarding the supernatant afterward. The cells were resuspended with 200 µL of 4% paraformaldehyde (PFA) fix buffer, vortexed briefly, and incubated for 20 min at room temperature in the dark. After centrifuging at 500 × *g* for 5 min and discarding the supernatant, the cells were washed twice with 1×PBS by centrifugation at 500 × *g* for 5 min each time. The cells were then resuspended in 0.1% Triton X‐100 and incubated for 15 min at room temperature in the dark. Following another centrifugation at 500 × *g* for 5 min and discarding the supernatant, the cells were washed twice with 1×PBS by centrifugation at 500 × *g* for 5 min each time. The cells were resuspended in 1×PBS and incubated with the primary antibody in each 100 µL of cell resuspension. They were incubated for 45–60 min at 4 °C in the dark. The cells were then washed with 1 mL staining buffer by centrifugation for 5 min, discarding the supernatant. The cells were resuspended with diluted fluorochrome‐conjugated secondary antibody in 100 µL 1×PBS, following the recommended concentration for secondary antibody dilution, and incubated for 45–60 min at 4 °C in the dark. Finally, the cells were washed with 1×PBS by centrifugation at 500 × *g* for 5 min, the supernatant was discarded, and the cells were resuspended in 1×PBS and analyzed on a flow cytometer.

The sample was then read on the cytometry (Attune NXT flow cytometry by ThermoFisher) at a flow rate of 100 µL min^−1^. A total number of 10 000 of events were collected using logarithmic amplification. Data acquired was then analyzed using Kaluza 2.0 software.

### Antibodies

Antibodies used for flow cytometry assay were obtained commercially as follows: anti RhoA antibody (Invitrogen, Waltham MA, USA (MA1134), and Rac1 antibody (Invitrogen, MA532928). Secondary antibodies used in the study include anti‐mouse 488.

### Cell Viability Assay

The viability of cancer cells and control cells was accessed by CCK‐8 from Abcam (Cambridge, UK). The cell suspension was added to a 96‐well plate. The plate was pre‐incubated in a humidifier incubator (37 °C, 5% CO_2_). Following the manufacturer's instructions, 10 µL of CCK‐8 solution was added to each well, and the plate was incubated for 3 h. The absorbance was then measured at 450 nm using a microplate reader.

### Statistical Analysis

Statistical analysis for all experiments was performed by GraphPad Prism software (Version X). Multigroup individual datasets with normal distribution were compared using a one‐way analysis of variance (ANOVA). Data are presented as the mean ± standard error of the mean for three technical replicates. RhoA and Rac1 concentrations were normalized to cell count. A Student's *t*‐test was used to determine *p*‐values, and results were considered statistically significant if the *p*‐value was less than 0.05. The exact number of replicates is given in each figure legend. Significance in all figures is denoted as follows: ^*^ *p* < 0.05, ^**^ *p* < 0.01, ^***^ *p* < 0.001, ^****^ *p* < 0.0001.

## Conflict of Interest

The authors declare no conflict of interest.

## Author Contributions

S.Y and N‐T.N conceived, designed the study and wrote the paper. N‐T.N provided the funding acquisition and supervision. S.Y, S.K, A.L, and N‐T.N performed the research and carried out the data analyses. S.Y, S.K, M.A.B, C‐T. L, A.L and N‐T.N edited the paper.

## Data Availability

Data available on request from the authors.
